# Supplementary material for: Hybrid Models and Biological Model Reduction with PyDSTool
Source: PLoS Comput Biol. 2012 Aug 9;8(8):e1002628. doi: 10.1371/journal.pcbi.1002628 (PMC3415397; doi:10.1371/journal.pcbi.1002628)
Supplement: Text S4 — Complete source code for the PyDSTool package (version 0.88.120504). Includes API documentation and help files linking to web pages. This file is identical to the current public release on Sourceforge.net. (ZIP) [file pcbi.1002628.s004.zip › PyDSTool/html/PyDSTool.Toolbox.optimizers.optimizer.standard_optimizer-pysrc.html]

xml version="1.0" encoding="ascii"?


PyDSTool.Toolbox.optimizers.optimizer.standard\_optimizer


| Home | Trees | Indices | Help | PyDSTool |
| --- | --- | --- | --- | --- |

Package PyDSTool ::
Package Toolbox ::
Package optimizers ::
Package optimizer ::
Module standard\_optimizer

[hide private]

[frames] | no frames]

# Source Code for Module PyDSTool.Toolbox.optimizers.optimizer.standard\_optimizer

```
 1   
 2  # Matthieu Brucher 
 3  # Last Change : 2007-08-10 23:13 
 4   
 5  """ 
 6  A standard optimizer 
 7  """ 
 8   
 9  import optimizer 
10   


11 -class StandardOptimizer(optimizer.Optimizer):


12    """ 
13    A standard optimizer, takes a step and finds the best candidate 
14    Must give in self.optimalPoint the optimal point after optimization 
15    """ 


16 -  def __init__(self, **kwargs):


17      """ 
18      Needs to have : 
19        - an object function to optimize (function), alternatively a function ('fun'), gradient ('gradient'), ... 
20        - a way to get a new point, that is a step (step) 
21        - a criterion to stop the optimization (criterion) 
22        - a starting point (x0) 
23        - a way to find the best point on a line (lineSearch) 
24      Can have : 
25        - a step modifier, a factor to modulate the step (stepSize = 1.) 
26      """ 
27      optimizer.Optimizer.__init__(self, **kwargs) 
28      self.stepKind = kwargs['step'] 
29      self.optimalPoint = kwargs['x0'] 
30      self.lineSearch = kwargs['line_search'] 
31   
32      self.state['new_parameters'] = self.optimalPoint 
33      self.state['new_value'] = self.function(self.optimalPoint) 
34   
35      self.recordHistory(**self.state)

36   
37   


38 -  def iterate(self, forceDir=None):


39      """ 
40      Implementation of the optimization. Does one iteration. 
41      (Optional) Provide known direction to overide step call in 'forceDir'. 
42      """ 
43      self.state['old_parameters'] = self.optimalPoint 
44      self.state['old_value'] = self.state['new_value'] 
45   
46      if forceDir is None: 
47        step = self.stepKind(self.function, self.optimalPoint, state = self.state) 
48      else: 
49        self.state['direction'] = forceDir 
50        self.state['gradient'] = -forceDir 
51        step = forceDir 
52   
53      self.optimalPoint = self.lineSearch(origin = self.optimalPoint, 
54                                          function = self.function, 
55                                          state = self.state) 
56      try: 
57        pest = self.function.pest 
58      except AttributeError: 
59        new_pars = self.optimalPoint 
60      else: 
61        # this would include self.optimalPoint if the lowest was found by the linesearch 
62        new_pars = pest.pars_dict_to_array(pest.log[pest._lowest_res_log_ix].pars) 
63        print "*** CHOSE pars with residual %.8f" % pest.log[pest._lowest_res_log_ix].residual_norm 
64      self.state['new_parameters'] = new_pars 
65   
66      self.state['new_value'] = self.function(new_pars) 
67   
68      self.recordHistory(**self.state)

69
```

  


| Home | Trees | Indices | Help | PyDSTool |
| --- | --- | --- | --- | --- |

|  |  |
| --- | --- |
| Generated by Epydoc 3.0.1 on Fri May 4 15:24:13 2012 | http://epydoc.sourceforge.net |
